# World’s Columbian Dental Congress—1893

**Published:** 1894-02

**Authors:** J. M. Walker


### THE DENTAL REGISTER.

VOL. XLVIII.] FEBRUARY, 1894. [No. 2.

### SOCIETY PROCEEDINGS.

### World's Columbian Dental Congress—1893.

SCIENTIFIC WORK BY SECTIONS.

Reported for the DENTAL REGISTER by Mrs. J. M. Walker.

SATURDAY, AUGUST 19TH.—SIXTH DAY.—GENERAL SESSION.

(*Continued from page 28.*)

The meeting was called to order at 11:00 A. M., by the President, and the Secretary-General read the following resolution from the Executive Committee:

"*Resolved*, That the thanks of the members of the World's Columbian Dental Congress be extended to the Hon. C. C. Bonney, President of the World's Congress Auxiliary; Clarence E. Young, Secretary; and Eugene J. Hazard, Private Secretary to Mr. Young, for the courtesies shown us during our stay in Chicago."

The reports of the following committees were read by title, and will appear in the volume of Transactions of the Congress, viz: Committee on the History of Dental Legislation in This and Other Countries, Wm. Carr, M.D., D.D. S., New York, Chairman.

Committee on the Care of the Teeth of the Poor, by T. H. Parramore, D.D.S., Hampton, Va., Chairman.

Communication on the Dental Use of Cocaine, by Dr. Viau, of Paris, France.

The topic for discussion, "What Relation Shall Dentistry Hold to Medicine?" was then taken up.

Dr. J. D. Patterson, Kansas City, Mo., thought the most rapid progress had been made by dentists as an independent profession, without, however, detracting from the fact that dentistry is a specialty in medicine.

Dr. J. Y. Crawford, Nashville, Tenn., would prefer to discuss the question, "What Relation Does It Hold to Medicine?" That it is a part of the healing art there can be no question. He thought there ought to be a well-equipped section in dental and oral surgery in every medical organization in the country. By bringing together those who are earnestly working for the advancement of the healing art in general and men who are capable, from a dental standpoint, of impressing upon the medical profession the importance of dental surgery, we should get the full recognition of the idea that dentistry is of a piece with hygiene from beginning to end, and that the perpetuation of civilization depended largely upon it. Dentists should be more thoroughly prepared for the treatment of all facial injuries than many of them are.

The subject was passed after a brief discussion.

The report of the Committee on Prize Essays was presented, awarding to Dr. George Cunningham, of Cambridge, Eng., the prize for the best essay on Oral Hygiene, and recommending that the medal be awarded to him.

The President, with a few fitting remarks, pinned the medal on the lapel of Dr. Cunningham's coat, stating that it would be suitably engraved. The medal consisted of a gold bar to which was suspended a plate, the token being of solid gold throughout.

Dr. Cunningham very feelingly expressed his gratification at having received the prize.

Dr. Godon, Paris, France, and Dr. Florestan Aguilar, Cadiz, Spain, offered resolutions signed by the different foreign representatives, expressing their thanks to the different members and those connected with the Congress for the many courtesies received.

The total registration was announced as 116 foreign and 999 American—total, 1,115.

The Chairman of the Executive Committee having been called away,

Dr. J. Taft, Cincinnati, was called upon to address the meeting as the representative of the Executive Committee. He said the work done by the Executive Committee had necessarily entailed much anxiety and great labor. Under the most favorable circumstances there would be anxiety in the minds of those who had such a work in charge; there were adverse circumstances to overcome, some actual, some which threatened greater obstacles than really presented. In the main, these have all cleared away, and we have a grand and undisputed success, and a far greater attendance than was anticipated. Even the financial troubles that have swept over our country have not been sufficient to prevent the grand success which has been achieved. It was thought that from five hundred to eight hundred members was all that ought to be expected, in view of the financial troubles, but we have had between eleven and twelve hundred members present. We are gratified that a large number are here from other countries. I voice the feelings and the sentiment of every American when I say that it is a matter of great gratification that we have been permitted to welcome so large a number of our professional brethren from all over the world.

What will be the outcome of this great work? I look upon it as prophetic of increased interest and rapid progress of our profession. Who would have attempted fifty years ago, or even twenty-five years ago, to prophesy such advancement and progress as has been made in the profession? Who can tell what the progress and the attainments shall be in the next twenty-five or fifty years? We dare not prophesy, but we are justified in saying that there will be an upward and a forward movement, and a richer development than has been realized. New things will arise, perhaps in the near future. Let us work on. Let this be a stimulus for us to go forward and do more and more. The door of opportunity is open wider to-day than it has ever been, and we should enter and occupy to the fullest extent that which is before us.

Our sisters, our lady friends, have had a door open to them through which they have entered into this profession. Is this not prophetic of onward movement? People sometimes criticise the entrance of women into this profession, just as they caviled and criticised when women entered the medical profession years ago. I believe that the ladies of the profession will do work that the men have not done. They will look more to the hygienic aspect. They will have more sympathy for the children, and they will look more to the oral diseases of children, more to prophylactic and hygienic efforts that may be made for the rescue and preservation of the little ones from attacks of disease.

Ladies and gentlemen, when we look at what has been done here, when we remember the fact that a larger number and a higher effort has been made here during this week in behalf of dental science than ever before, we should be very proud. If another Congress seems proper, let us have it. The profession is able and capable of doing a great work, and it may be that a greater work than this will be accomplished in the future.

The President of the Congress, Dr. L. D. Shepard, in his closing address, briefly recounted the labors of each of the committees, to whose labors much of the success of the Congress was due, and expressed his appreciation of their faithful, efficient, unselfish discharge of the duties intrusted to them.

He said: It may be truly said that the work done in this Congress has been most extraordinary. Usually, when there are side-shows or outside attractions, people are taken away from the dry and less exciting professional work of the meetings; but, notwithstanding the marvelous attraction so near us, the meetings in this hall have been well attended, the clinic rooms have been thronged, and the Section rooms have been crowded. I think, under the circumstances of the extraordinary counter-attractions, the attendance of so large a number of members at the sessions deserves special mention, and shows that they came here for dentistry first, and have attended to it, while they were here. The very wise and thoughtful provision made for carrying on the work, by those in charge, is the cause of the success of this Congress.

While no one could be more conscious of the honor which this position brings with it, there is also the consciousness that there are many other American dentists who have done infinitely more for the advancement of this country, and of the world. We have members who are known throughout the world as original, scientific investigators, and their names will live in history, not as presiding officers, but as workers in the fundamental fields of research which make true progress. The President is in this Congress but a figure-head for the expediting of the transaction of the business which has been planned by others, and he desires personally to thank every member for the kindness he has received at their hands.

The friendships which have been made here will last through life. We have met the eminent men of the profession from almost every clime; and friendship, the result of personal meeting and personal converse, is an immense factor in progress. For the kind resolutions which were passed in behalf of the American members of the Congress, we return our thanks that those who have come from afar have enjoyed themselves, and can tell us truly that while the record has been passed of the biggest meeting, the more valuable record of the largest work of progress has also been made.

We appreciate the kindly words of our foreign friends, and trust as they go to their homes that success will attend them, and as the American dentist, who is a great traveler, shall visit their homes, we know that the warmest kind of welcome will await him at their hands.

Gentlemen from abroad, we are glad you came. We have enjoyed your company, and the bright thoughts and valuable instruction which you have brought to add to what has been gathered from our own shores, and we heartily indorse the movement that in future an exigency like the present shall be the motive for the formation of another World's Dental Congress.

There is nothing now for me to do but, by the authority conferred in me, to declare this World's Columbian Dental Congress adjourned *sine die.*

At the conclusion of the session, a photograph of the gathering was taken in the hall, after which the members separated, and the World's Columbian Dental Congress was at an end.

In addition to the eight sections, the daily work of which has been recorded, what might be termed Section 9, "Histology and Microscopy," held two night sessions, with lantern exhibits.

On Wednesday evening, Dr. R. R. Andrews, Cambridge, Mass., delivered a lecture, illustrated by photo-micrographs, entitled, "A Contribution to the Study of the Development of the Enamel."

This paper embodies the result of Dr. Andrews' investigations subsequent to the paper presented by him before the Dental Section of the Tenth International Medical Congress at Berlin, in 1890.

He said: My studies since then lead me to believe that there exists in the young developing enamel, something that has the appearance of fibers, guiding and sustaining the globules that are excreted from the enamel-cells which are to form the future enamel-rods, upholding and supporting them.

After citing the views held by John and Charles Tomes, Dr. J. L. Williams, Prof. W. X. Sudduth, Kolliker and Klein, he said: Some little time after finishing my former paper on "Enamel, Its Development and Calcification," I read it to Prof. E. L. Mark, of the Biological Department of Harvard University. He stated that I had found and demonstrated new points about the enamel that a German investigator had also recently described; that both papers gave similar views. At my request he translated the paper from the German journal in which it was published. Its title was, "On the First Processes of the Deposition of the Enamel," by Dr. Graf Spee. The description he gave of this process was so nearly like my own, that I read it with considerable surprise.

I did not know that any one had described the enamel-rod as being formed by minute globules coming through the cell. But he had seen these minute and highly refractive globules in the body of the cell, and says that when the tissue is properly prepared—and he lays great stress on this point—they are always to be found there at the time of the formation of the enamel. Their entire absence at earlier stages is an indication that these globules are an enamel substance. He gives to them the name

"enamel-drops," and says he saw these "enamel-drops," when enamel is to be formed, appear only in the half of the enamel-cell which rests on the dentine; afterward, farther up in the cell, but not quite up to the region of its nucleus. Dr. Spee's "enamel-drops" were really what I described as minute calco-spherites, which, merging together, had formed large globules, of a substance which I believed to be calco globulin.

He spoke of a paper by J. Howard Mummery, entitled, "Some Points in the Structure and Development of Dentine," read before the Royal Society, March 5, 1891, saying, "appearances noted and demonstrated by Mr. Mummery in this paper recalled to my mind similar appearances which I had seen in the developing enamel, appearances that I could not then explain, that I did not understand. As some of his results will be of interest to us, while considering this subject, I shall try and give a brief idea of them."

After summarizing Mr. Mummery's paper, and describing the processes by which the specimens to be presented had been obtained, he said: To sum up my conclusions: I am led to believe that there probably exists in developing enamel, as has already been found in developing bone and dentine, a fibrous sub-structure on and between which the enamel is deposited. After the enamel is wholly formed, its existence seems to be wholly blotted out in the dense calcification of the tissue. In sections of wholly-formed enamel, I have never been able to trace it, although I have tried the methods of those who claim to have seen it. In regard to a beaded protoplasmic reticulum of living matter in formed enamel, I have never been able to find it. I believe with Klein, that it is improbable that nucleated protoplasmic masses are contained in the interstitial substance of the enamel of a fully-formed tooth.

This paper was passed without discussion, and was followed by a lecture by Dr. W. X. Sudduth, Minneapolis, Minn., also illustrated by lantern slides, "On Some of the Forces that Influence the Form of the Jaws and Teeth During the Process of Development."

In discussing the subject of the evolution of the teeth it is essential that there shall, at the outset, be a full understanding regarding the relationship of tooth and jaw, as the environment of the tooth has much to do in its formation.

A tooth in its early stages of development is wholly composed of soft tissues, and lies in a bed of embryonal connective tissue, constituting the fœtal jaws.

This easily compressible matrix is bounded on the outer side by the cheeks and lips which tend to compress the jaw and hold it firmly against the tongue. The shape of the fœtal jaw depends, to a very great extent, upon the form of the tongue, a pointed tongue giving a pointed arch, while a blunt tongue will give a correspondingly shaped one. From this it will be seen that even before there is any osseous development the form of the arch is, to a great extent, determined by the environment of the jaws.

Before the tooth assumes definite form ossification of the maxillæ begins, and each germ is separated into its own alveolus, so that even as early as the third month of gestation the type of the jaws is established. The development of bone in the jaws proceeds in the substance of the soft, connective-tissue matrix in such manner as not to disturb the form of the arch established in the first month of gestation.

The successive stages of tooth development and the influences guiding their respective forms were traced to the time of eruption and root-formation. He said, anything that interferes with the normal process of development will necessarily leave its impress on the crown in the shape of malformations. This is so well known that it need not be discussed in this connection. Suffice it to say that congenital syphilis, rachitis, scrofula, all the exanthemata and pathological processes, in connection with the absorption of the roots of the temporary teeth, are among the several factors that pathologically influence the form of the crowns of the teeth. Faulty nutrition, from whatsoever cause, results in poorly calcified and malformed teeth in a great variety of forms.

The effect of use and disuse, and the indirect influence of environment were next considered, and the changes which may and do occur in the form of the teeth as the result of the action of the law of "variation," whereby additional cusps are formed and other similar changes which are subject to inheritance.

A portion of the lecture was given to the change in form of the jaws as the result of artificial selection in animals under domestication, where the position of the teeth in the jaw as well as change in form and even suppression in the number of teeth result, the most marked being the common swine and pug dog.

A series of slides were shown in illustration of the lecture.

Dr. M. H. Cryer spoke of the effects of breeding for "short-faces" upon the pug dog, showing that the teeth became exceedingly irregular, the lower jaw protruding beyond the upper, producing the condition known as "underhung." Their anterior teeth are soon lost for lack of use through non-occlusion, and the posterior molars often are not erupted, and upon dissection of the jaws are found in a rudimentary condition.

At the Thursday evening session Dr. D. E. Caush, of Brighton, Eng., read a paper entitled, "Some Changes that Take Place in and Around the Pulp-Canal."

He said: During the microscopic examinations of exostosed human teeth, my attention was continually drawn to certain alterations in the shape and structure of the pulp-canals; it was an unusual thing to find in an exostosed tooth a perfect pulp-canal, and this led me to the examination of a large number of these teeth. The subject has been under observation about five years, and the deductions drawn are from the examination of between two and three thousand teeth.

The changes noted by the author were various irregularities of outline in the pulp-canals, until, in some cases, the whole of the original outline of the pulp-canals had disappeared, leaving in its place a very irregular canal. The canal was also much enlarged in some specimens, while in others the excavations had extended until a second canal had been formed, frequently passing at right or acute angles to the original canal, continuing until it passed through the dentine into the cemental tissue. These canals were either simple or branched, or instead of canals passing thus at angles to the pulp-canal, the two or three canals of an inferior or superior molar may be united together into one irregular canal.

In all cases where this change has taken place the margins of the canals show a more or less irregular edge, produced by the semilunar excavations of the dentine. A second change more marked even than that of the excavation oftentimes follows, for in these depressions it is not at all unusual to have a deposition of new bony tissue entirely different in microscopic structure from the dentine which surrounds it. In this new tissue we have no tubuli radiating to a given center, as in the ordinary dentine, but in place of the tubuli we have a number of lucunæ and canaliculi, in character somewhat like those found in true cemental tissue.

The irregular margin and semilunar character of the edges give a clue, as to how these changes are brought about, showing that there must have been absorption going on in a greater or lesser degree, according to the amount of change that has taken place.

It is evident (microscopically) that such an edge could not be produced by the addition of new tissue alone, and in cases where secondary dentine is deposited, either as dentine of repair, or as a result of pulp-calcification, for in either of the cases the microscopic structure of the tissue is quite different; in all these cases the new tissue is always added directly to the older tissue without any excavations or absorptions of the original margin, and the secondary dentine is deposited directly from the odontoblastic layer of the pulp. On careful examination of the exterior surface of an exostosed tooth, the first thing to which our attention will be drawn is the thickened condition of the membrane, and it may appear two or three times as thick as it would appear in health, and at certain places under the membrane we may frequently find excavations going on and giant-cells in position; again, in another place in this root we may find some older excavations, and a layer of new tissue filling up the excavations and increasing the size of the exostosed tooth by the additional tissue formed, in fact, the three stages of the development of cemental tissue in exostosis.

The initial point in these changes is, the layer of odontoblasts or lining cells of the pulp-canal, the source of nourishment from the pulp to the dentine. It is from this layer that the secondary dentine is formed, but under certain circumstances the character of the cells forming this layer changes, both in shape and size. Thus, if by such cause as congestion, or slight inflammation of the pulp a greater amount of blood is brought the blood-vessels in the pulp, these cells appear to lose their original shape, and enlarge, the nuclei become active, and new cells are rapidly formed by cell-division. As these new cells continue to form, a layer like the alveolar dental membrane in appearance is produced, and this newly-formed layer presses upon the dentine. The cells in juxtaposition with the dentine begin to absorb the latter, and thus obtain more room for further development. This goes on as long as there is an abnormal blood-supply to the pulp, and thus owing to either a long or short time of pressure upon the dentine, caused by the excess of formative material brought to the cells by the increased blood-supply, we may have many or few excavations produced, as in the case of chronic inflammation of the pulp, we get many excavations, and oftentimes these excavations penetrate more deeply into the original tissue.

Should the tooth be extracted about this period, nothing but the excavations or the thickened membrane will appear in the pulp-canal. If the tooth is not extracted, the whole of the pulp becomes more or less congested and altered in character; the odontoblastic layer being destroyed, there is little or no intercommunication with the dentine by the tubuli, and at this stage one of two things may occur:

1. The blood-supply becomes normal, the congestion temporarily passes away, and for a short time the tooth is tender to the touch, as if there had been periostitis, and from this stage it gradually becomes comfortable, and remains so until the inflammation again commences, or—

2. The giant-cells change their character, and become formative cells, producing as a result of calcification, a fresh tissue in the canal, in character and in microscopic structure resembling cementum, developed also from a membrane similar in character to the alveolar dental membrane.

This paper was illustrated by a number of slides, showing the irregular outlines of the canal, the excavations spoken of, the lateral canals, etc.

DISCUSSION.

Dr. G. V. Black said: It has fallen to my lot to cut a great many of these sections, and to study them and to write upon them, to some extent. Now, there is a number of different calcifications, if I may use that term, occurring in the pulp-chamber. We have the simple dentine, as shown; then, again, occasionally the true dental tumor, growing out of the wall into the pulp-chamber, presenting the canaliculi. The thing we find the most of, and the thing that seems to have been shown mostly upon the screen, is calcification that has occurred as degeneration, which is undoubtedly, to my mind, simple degenerate calcification, such as we have in the arteries, and around the valves of the heart and other parts of the body. These occur very frequently in the pulp-chamber, when, from any cause, either from age or from disease, or abrasions of the teeth, calcification of these organs occurs. This may be attached to the pulp-chamber, as we have seen upon the screen, or may be free within the pulp in various forms; they are not properly tissues, they are simply calcifications of the tissue in a state of degeneration.

Now, as to absorption, some of those long lines running through the dentine, that we have seen on the screen, I am in the habit of determining as blood-vessels included in the dentine. We find these canals shooting out in different directions from the pulp-chamber, and they certainly are the things I have been in the habit of interpreting as forming the vessels in these lateral canals. It is exceedingly rare. We have them abnormally large, and filling up with calcific matter. We may occasionally have them filling up by cementum, and that is rather rare; but the true cementum, having the true characteristics, is a rare thing indeed.

Dr. A. O. Hunt, Iowa City, Ia., said that, in his opinion, all deposition within the body of the pulp would be of a character like cementum, rather than dentine, for the reason that the blood-supply for sustaining it comes directly from the dental membrane.

Dr. Geo. Cunningham, of Cambridge, Eng., read a paper entitled, "Luxation, or the Immediate Method, in the Treatment of Irregular Teeth."

The paper was a description of a number of cases of the immediate method of treating irregularities by luxation, which has been practiced by a number of English dentists. When the slow mechanical means are out of the question, this method may be regarded as filling a niche not previously occupied by any other operative means at our disposal.

The paper was illustrated by a large number of slides, showing various cases related.

In reply to questions, Dr. Cunningham stated that the root of the tooth was moved as well as the crown, though in his opinion the pulp was not capable of being stretched to any great extent. Also that more force was required in this operation than for extraction.

Dr. Jarvie said of this method: He has shown some marvelous results, in which he has moved teeth a quarter of an inch, with the pulps kept alive, and, barring the pain of the operation at the moment, no results that are severe to the patient. Now, if irregularity exists, and the teeth can be made regular by a method that will involve only a few moments pain, it is infinitely better than one that extends over several weeks or months.

Dr. Cunningham further said: In regard to the comparative merits of the slow and the quick methods, I do not consider that I am in a position yet to give a definite opinion. It has been a long time since the first operation, as you will observe, and this is the first time I have brought it before a body of this kind. I have a certain amount of diffidence in bringing forward so simple and easy a way for both the patient and operator. I am not in a position to say that such an operation replaces the older method. I only claim that in these operations I have taken them where the ordinary operations have failed. The slower method is the surer, and at the same time there is an element of hope in this operation which is very great indeed.

Dr. Jarvie: Occasionally cases present themselves in which I wish I could do what Dr. Cunningham has done so successfully, but I have, every time, dreaded to attempt it.

Dr. Cunningham: I am ready to perform the operation at any time, without fear, when the patient has arrived at adult age. Adjourned.

EXHIBITS.

Among the exhibits made at 102 Michigan avenue, in connection with the Dental Congress, were the following:

Dr. Barrie, Paris, France, exhibited a hot-air injector with four adjustable points for puncturing abscesses, injecting abscesses, enlarging fistulæ, and for ablation of epulitic growths. The instrument is attached to a reservoir containing gasoline, to which there is a bulb attached for forcing air through the reservoir. In use, the point is heated over the flame of an alcohol lamp or Bunsen burner, the heat being kept up by the combustion of gasoline vapor. It is very convenient for drying out teeth or any other purpose to which a cautery may be put.

An electric mallet devised by E. Bidaud, of Paris. This mallet has the interrupter so arranged that closure of the circuit is made when the plugger-point comes in contact with the filling. This secures the delivery of a single blow at the moment when applied, as in the Snow & Lewis automatic, and not a continuous series of rapid blows, as in the ordinary electric mallet. The force of the blows is controlled and graduated from light to heavy at will.

An electric stomatoscope, in which illumination from a small electric lamp, encased in a hard rubber cylinder, is projected through a glass rod, the free terminal end of which, about five to eight centimeters in length, is ground hemispherically in shape, and so acts as a strong convex lens. The light when projected through this rod gives a powerful illuminating effect, and several of these rods, with various curvatures, are provided, so that all parts of the oral cavity, and in fact any of the cavities of the body may be illuminated thereby.

The great advantage of this instrument is the absence of any danger of breakage, or of any appreciable rise of temperature, at the point where the light is delivered.

The universal forceps devised by Dr. Poinsot, of Paris, with changeable beaks. This instrument departs widely from the usual form, as the beaks are not closed by the leverages on the handle, but by a mechanism similar to a monkey-wrench.

Dr. Godon's exhibit of so-called "Immediate Prosthesis" originally introduced by C. Martin, of Lyons, France, was one of the extremely interesting exhibits. One was a hard-rubber piece to substitute for a portion of a jaw and teeth which had been resected on account of disease. This piece was made before the surgical operation, measurements having been made for size and shape. Immediately after the operation the piece was placed in position and screwed to the remaining portion of the jaw. It was simply a temporary appliance to prevent the falling in of the parts where the muscles are inserted, and after being worn a few months, its object being accomplished, it was removed and replaced with a hard-rubber plate and teeth of similar form.

Also soft-rubber vela to be attached to hard-rubber plates for closing fissures in the soft palate. In constructing this apparatus, he dissolves rubber in chloroform and paints it over his matrix or mold. In this way he avoids air-spaces and consequent porosity after vulcanization.

In one case the velum is hollow and subsequently partially filled with water, to make it more adaptable to the soft parts. This is done by vulcanizing it over a plaster core; then the piece is punctured at some point and the plaster removed through the opening, and partially filled with water and closed by means of a disk or washer and screw, by which also the attachment is made to the plate.

Dr. Godon exhibited an artificial tongue made of soft rubber, filled with water. This can be introduced after an operation necessitating the removal of the tongue; it can be slightly used and controlled by the remaining stump.

Dr. Godon presented also several other appliances for removable bridge-work, etc.

Dr. Geo. B. Clement showed a series of slides under the microscope which, he claims, proves that pyorrhœa alveolaris is caused by the calcic infiltration of the cementum, producing calcic hypostasis. His theory is that the cementum while it receives just so much nourisnment as will allow it to retain its natural condition will be healthy, but in case of super-nutrition a condition of inflammation will occur, while in the opposite condition the microscopical vessels of nutrition in the cementum become obliterated by the deposition of lime-salts, and the disease pyorrhœa alveolaris is the result. His specimens showed sections of cementum from healthy and diseased teeth, and seemed to prove the truth of the theory.

Dr. J. W. Penberthy exhibited a new bracket-table. The peculiarity of this table consists in the arrangement of the bottom of the drawers. These are hung on pivots, so that when the drawer is pulled out the back end of the bottom swings down. Holes extend through the bottom to contain the instruments, and when the drawer is extended the points of the instruments are in sight and convenient to the hand.

Dr. J. H. Morrison, of Connersville, Ind., showed a system of punching up crowns from solid plate. By forcing a disk of metal through different-size holes he gets a parallel-sided tube closed on the end. He then about half fills this tube with small shot, places it upon the die-plate representing the coronal surface he desires, and with repeated blows of the hammer the cap is made to take the form of the masticating surface of the tooth. The spreading of the lead gives a bulging shape to the part below the crown.

Dr. Geo. D. Sitherwood, of Bloomington, Ill., exhibited a closed-top oak work-table, suitable for the operating room, for the appliances and materials needed in crown and bridge-work.

This had a roomy space, which accommodated an oxyhydrogen blow-pipe and a porcelain furnace. This part could be closed up like a desk whenever desired. Underneath were two drawers of convenient size.

He also showed specimens of aluminum dental plates.

Dr. C. W. Jones exhibited a root-trimmer and facer for trimming the roots, without lacerating the gum, for there ception of a collar or crown.

Dr. V. H. Jackson showed a large number of casts, fitted with regulating appliances, as described in his paper read before Section 7.

Dr. L. E. Custer exhibited an electrical cabinet for the electrical mallet, hot-air syringe, illuminator, etc.

Dr. J. Austin Dunn exhibited the "Dunn Syringe and the Dunn Hand Matrix."

Dr. H. N. Young exhibited drawings and casts of the jaws of a case of *osteitis deformans*, or *acromegaly*.

Dr. Richards, of Knoxville, exhibited a set of artificial teeth encrusted with young oysters, which were found in an oyster-bed on the coast of North Carolina.

Among other articles exhibited were the following: The Hollingsworth Crown and Bridge System and Appliances, The Berry Electrical Dental Engine, a collection of instruments manufactured in Japan, with the S. S. W. trade-mark on them, Buxbaum's Dentimeter, Pyrozone, by McKesson & Robbins, Harcourt's Dental Engine.

Dr. Geo. Cunningham, London, England, showed a very interesting collection of small muffles and crucibles suitable for his new low-fusing continuous gum.

He also exhibited some artificial teeth, which, by means of the low-fusing body, he had tinted in different shades, showing the value of the process in obtaining the most artistic results in teeth bought at the depots, which were all that could be desired, as to size and shape, but which, in color, lacked the individual characteristics demanded of the case.

Another use for this material was shown in an artificial crown, where the connection between the porcelain tooth and metallic cap was made by the fused body. Pitting, grooving and otherwise marking of the depot teeth in imitation of natural ones in particular cases were exhibited, showing very artistic effects.

There was on exhibition an interesting collection of different pieces of workmanship by Paul Alfred Koelliker, of

Zurich, Switzerland, the most important being an obturator adapting itself to all movements of the soft palate, with gold hinge attachment to a vulcanite plate, a most beautifully executed contrivance.

There were two or three curious specimens of prosthetic work made in Dr. Koelliker's earlier experience. One was a full lower denture made on a base carved from the tusk of a hippopotamus, with natural teeth inserted ; put in the mouth in 1855 and worn continuously until 1867, when it came into his possession upon the death of the patient. Another was an upper denture with English tube teeth in front on a gold plate and hippopotamus blocks for the molars. This piece was put in the mouth in 1870, and is very skillfully done.

Another curiosity in the collection was a partial upper and lower set made of gold and connected by spiral springs, the upper to the lower. It had natural teeth attached, also teeth carved from the hippopotamus, both showing decay which had occurred after being placed in the mouth ; made about 1830.

### DEMONSTRATIONS.

In connection with the clinics at 102 Michigan Avenue, in connection with the Dental Congress, the following demonstrations were given :

Dr. Geo. Cunningham, London, England, showed his process of continuous-gum work with low-fusing enamel and body. Also the staining or tinting of artificial teeth to resemble accidental defects in natural teeth.

Dr. Sitherwood : A method of fusing porcelain around the band of a porcelain crown, which would insure perfect joints and, with proper shading, an artistic result.

A swaged plate of pure aluminum was also shown, and the method of annealing and swaging described.

Dr. Broadbent, Chicago : Bleaching a left superior incisor badly discolored for about a year and markedly affected by pyorrhœa alveolaris. The treatment consists in applying pyrozone, 25 per cent., outside by means of a spray, and on cotton within the pulp-canal.

The pyrozone was applied and rapidly volatilized by means of an air spray.

Dr. C. E. Blake, of California, demonstrated the use of "Muriatic Ether," (ethyl chlorid), as a local anæsthetic, and the use of his new gum-cutting and extracting forceps.

Dr. C. Sill, of New York City, demonstrated the use of vaseline to allay the irritation from the use of oxyphosphate and oxychloride cements. He mixes it with the powder in the proportion of fifteen parts oxide of zinc to one of vaseline, grinding up together carefully. It absolutely prevents the irritation, and his experience teaches him that the usefulness of the filling is in no way impaired.

Dr. W. H. Marshall, Oxford, Miss.: "The Marshall Matrix," for amalgam fillings.

Dr. C. C. Carroll inserted an aluminum amalgam filling, demonstrating the use of the Carroll-Campbell Engine and Handpiece.

Dr. T. W. Brophy demonstrated the use of the "Doriot Surgical Engine" in an operation for the removal of a tumor.

Dr. L. C. Bryan, of Basle, Switzerland, demonstrated the use of his forceps for the immediate correction of irregularities. The tooth operated upon was a right superior lateral. The operation consists in cutting the process around the tooth to be moved, then placing a stout piece of metal the shape of the exterior of the arch around the teeth, and with the forceps moving the tooth up to the line, and ligating it in its correct position, local anæsthesia being produced by the injection of cocaine.

Dr. W. G. A. Bonwill demonstrated the use of his improved mechanical mallet. He also gave a demonstration, in his own mouth, of his method of clasping teeth on all gold plates, from one to full upper set, where one tooth only remains.

He explained his principle of practice in the use of pink gutta-percha base plate for filling all cavities, and obtaining greater width of separation between the bicuspids and all the molars. He showed its use as a matrix in filling with gold or amalgam, also the use of paraffin in being melted over and into all oxyphosphate fillings to make them harder and last longer; and also melted it over all-gold fillings to hermetically seal any vacancy that might be between bone and filling.

Dr. Thos. Fillebrown demonstrated the method of sustaining ether-anæsthesia by the use of blow-pipe and foot-bellows. The claim is made that anæsthesia may be thus kept up for a long time without unpleasant or injurions effect upon the patient.

He also gave a demonstration of his methods in hypnotic suggestion in the painless extraction of teeth.

Dr. J. J. Stedman, Laporte, Ind., demonstrated his method of retaining full dentures by means of the "Stedman Springs."

Dr. Schreier exhibited and explained his method of treating putrescent pulps with Natrium Kalium.

Dr. C. C. Carroll demonstrated practically his cast aluminum work.

Dr. O. B. Love, San Antonio, Texas, showed the use of mouth illuminator.

Dr. Louis Jack, Philadelphia, demonstrated the use of the "Jack Matrices and Matrix Pluggers."

Dr. W. H. Richards demonstrated his method of filling tortuous root-canals at the apical foramen with metallic fiber.

Dr. Geo. W. Weld, New York, demonstrated the non-irritant action of his Syrup of the Chloride of Iron.

---

Diana Clifford Kimber will soon publish a text book on "Anatomy and Physiology for Nurses," in connection with Louise Darche. Miss Kimber's experience as assistant superintendent in both the New York City and the Illinois Training School for Nurses has led her to feel the need of such a manual and to undertake the work. It is designed to fill a middle place between the text-book written for medical students and that for use of children in schools. The subject is presented in a scientific manner, but the technicalities which discourage the average student have been, so far as possible, avoided.

